# Epley-Type Maneuvers Versus Brandt-Daroff Exercises for Posterior-Canal Benign Paroxysmal Positional Vertigo: A Systematic Review and Meta-Analysis

**DOI:** 10.7759/cureus.112282

**Published:** 2026-07-08

**Authors:** Fahad Alkhalefah, Waleed Alhazmi, Faisal Alkhalefah, Musaad Alawaji, Anas Almutairi, Alwaleed Alotaibi

**Affiliations:** 1 College of Medicine and Medical Sciences, Qassim University, Unaizah, SAU; 2 Department of Otolaryngology, Qassim University, Buraidah, SAU; 3 College of Medicine, Qassim University, Buraidah, SAU

**Keywords:** benign paroxysmal positional vertigo, brandt-daroff exercise, canalith repositioning, epley maneuver, meta-analysis, posterior canal, recurrence, systematic review

## Abstract

Posterior-canal benign paroxysmal positional vertigo is commonly treated with canalith repositioning maneuvers or habituation exercises. Epley-type maneuvers may provide faster symptom and nystagmus resolution than Brandt-Daroff exercises, but comparative effects on recurrence, functional outcomes, and adverse events remain less certain. This systematic review and meta-analysis synthesized direct comparative evidence evaluating Epley-type maneuvers versus Brandt-Daroff exercises in adults with posterior-canal benign paroxysmal positional vertigo. PubMed/MEDLINE, Web of Science, ScienceDirect, and Cochrane Central Register of Controlled Trials (CENTRAL) were searched using terms related to benign paroxysmal positional vertigo, Epley-type or canalith repositioning maneuvers, and Brandt-Daroff or habituation exercises. English full-text comparative studies were included when they enrolled patients with posterior-canal benign paroxysmal positional vertigo and reported extractable clinical outcomes. Binary outcomes were pooled as risk ratios using random-effects models, while continuous outcomes and adverse events were summarized narratively because of heterogeneity in reporting. Thirteen studies involving 911 analyzed participants were included in the qualitative synthesis. Six studies involving 348 participants contributed to the main short-term treatment-success meta-analysis. Epley-type maneuvers improved short-term treatment success compared with Brandt-Daroff exercises (risk ratio 2.10, 95% confidence interval 1.43-3.07; I² = 65.6%). Five studies involving 301 participants contributed recurrence data. Recurrence did not clearly differ between groups (risk ratio 0.91, 95% confidence interval 0.60-1.36; I² = 15.3%). Functional outcomes generally improved after both interventions, but differences in scales, timing, and reporting precluded meaningful pooling. Reported adverse events were generally minor and transient. Epley-type maneuvers are associated with better short-term resolution of posterior-canal benign paroxysmal positional vertigo than Brandt-Daroff exercises, but available evidence does not show a clear recurrence advantage. The certainty of evidence was low for short-term success and recurrence and very low for adverse events, mainly because of risk of bias, heterogeneity, imprecision, and inconsistent reporting.

## Introduction and background

Benign paroxysmal positional vertigo (BPPV) is a common peripheral vestibular disorder characterized by brief episodes of positional vertigo with characteristic positional nystagmus. The posterior semicircular canal is the most frequently involved canal, and diagnosis is usually established clinically with the Dix-Hallpike maneuver, supported by the presence of typical positional nystagmus and compatible symptoms [1-4].

Physical maneuvers are the mainstay of posterior-canal BPPV management. Epley-type maneuvers, including the standard Epley maneuver, modified Epley maneuver, self-Epley maneuver, particle repositioning maneuver, and canalith repositioning procedure, aim to relocate otoconial debris from the posterior canal back into the utricle. Brandt-Daroff exercises are repeated habituation/home exercises that can be performed by patients and may be useful when supervised maneuvers are not feasible, when home therapy is preferred, or when access to specialist care is limited [1-13].

Mechanistically, posterior-canal BPPV is usually explained by displaced otoconia from the utricle entering the posterior semicircular canal, where gravity-dependent head movement produces abnormal endolymph movement, cupular deflection, positional vertigo, and nystagmus. Epley-type maneuvers aim to return this debris toward the utricle, whereas Brandt-Daroff exercises rely on repeated positional movements and habituation and are often used as a practical home-based option. Several included studies also frame these interventions within guideline-based clinical management, but guideline-level recommendations do not directly resolve the focused comparison of Epley-type maneuvers versus Brandt-Daroff exercises for short-term success and recurrence [3,10,12,13].

Although Epley-type maneuvers are generally regarded as highly effective for rapid symptom and nystagmus resolution, the comparative evidence against Brandt-Daroff exercises is heterogeneous. Some studies have reported a clear early advantage for Epley-type maneuvers, whereas others have found smaller or no clinically important differences between groups. In addition, some studies focus on immediate or short-term Dix-Hallpike conversion, while others report patient-reported disability, recurrence, or longer-term follow-up [1-13]. This creates an important distinction between early treatment success and long-term recurrence, which should not be interpreted as the same outcome.

Previous comparative studies have evaluated clinician-performed Epley maneuvers, modified Epley maneuvers, self-Epley maneuvers, particle repositioning maneuvers, and canalith repositioning procedures against Brandt-Daroff exercises in different clinical settings and follow-up periods [1-13]. However, a focused synthesis of direct Epley-type versus Brandt-Daroff comparisons remains clinically useful because these approaches differ in setting, supervision, patient burden, and suitability for home-based management. The present review, therefore, focuses on direct comparative evidence evaluating Epley-type maneuvers versus Brandt-Daroff exercises in adults with posterior-canal BPPV. The primary emphasis is on short-term treatment success and recurrence after initial successful treatment, with secondary synthesis of functional outcomes and adverse events.

## Review

Methods

Design and Reporting Framework

This systematic review and meta-analysis compared Epley-type maneuvers with Brandt-Daroff exercises in posterior-canal BPPV. The manuscript was structured according to PRISMA 2020 principles [14]. This review was not prospectively registered. Eligibility criteria and analysis decisions were finalized before the final quantitative synthesis.

Eligibility Criteria

Studies were eligible if they enrolled patients with posterior-canal BPPV and included a direct comparison between an Epley-type maneuver and Brandt-Daroff exercises. Eligible Epley-type interventions included the standard Epley maneuver, modified Epley maneuver, self-Epley maneuver, particle repositioning maneuver, and canalith repositioning procedure. Studies were included if they reported extractable clinical outcomes such as Dix-Hallpike conversion, absence of positional nystagmus, symptom resolution, recurrence, Dizziness Handicap Inventory scores, Vestibular Activities and Participation scores, other functional scales, or adverse events.

Studies were excluded if they were reviews, systematic reviews, meta-analyses, network meta-analyses, guidelines, case reports, or non-comparative studies, did not include a direct Epley-type versus Brandt-Daroff comparison, were not focused on posterior-canal BPPV, did not provide English-language full text, or lacked extractable relevant outcomes. Multi-arm studies were eligible when they contained both an Epley-type arm and a Brandt-Daroff arm. Choi et al. was summarized separately as cupulolithiasis-specific evidence because it enrolled posterior-canal cupulolithiasis rather than typical posterior-canal canalithiasis [10].

Information Sources and Search Strategy

Searches were conducted in PubMed/MEDLINE, Web of Science, and ScienceDirect from database inception to March 7, 2026. Search results were exported and deduplicated on March 16, 2026, using Digital Object Identifier (DOI), PubMed Identifier (PMID), and normalized title matching. Title and abstract screening were completed on March 16, 2026, followed by full-text retrieval and eligibility assessment. Data extraction, quantitative synthesis, risk-of-bias assessment, and certainty-of-evidence assessment were completed by May 6, 2026. Searches used three concept blocks: BPPV terms, Epley-type/canalith repositioning terms, and Brandt-Daroff/habituation terms.

Cochrane Central Register of Controlled Trials (CENTRAL) was searched as a supplementary update on May 9, 2026. The search produced 59 results and yielded no additional eligible included studies. Because export was not available in the accessed interface, results were screened manually within the Cochrane Library search interface, and the search history, result count, and candidate records were documented.

The primary search string was: ("benign paroxysmal positional vertigo" OR BPPV OR "positional vertigo") AND (Epley OR "modified Epley" OR "self-Epley" OR "canalith repositioning" OR "particle repositioning" OR PRM OR CRM) AND ("Brandt-Daroff" OR "Brandt Daroff" OR habituation). Database-specific search syntax was adapted from this core strategy according to the search functions available in each database. Citation chasing and hand-searching were used to identify additional relevant reports not captured in the database exports.

Study Selection

Database export files were deduplicated using DOI, PMID when available, and title matching. After deduplication, titles and abstracts were screened independently by two reviewers who were blinded to each other’s decisions. Full texts of potentially eligible reports were then assessed independently by the same two reviewers, with disagreements resolved by discussion and, when necessary, adjudication by a third reviewer. Studies were classified as included, excluded with reason, or not retrieved. A previously identified Soto Varela et al. 2001 report was classified as not retrieved because the full text could not be obtained [15].

Data Extraction

Data were extracted into an Excel sheet (Microsoft® Corp., Redmond, WA). Extracted variables included author, year, country, study design, setting, diagnostic criteria, BPPV subtype, inclusion and exclusion criteria, sample size, age, sex, intervention details, comparator protocol, follow-up duration, short-term success, recurrence, functional scales, adverse events, and extraction caveats. For multi-arm trials, only the eligible Epley-type and Brandt-Daroff arms were used in direct pairwise meta-analysis; other arms were retained for context but not pooled in the primary pairwise comparison. Data extraction was performed by one reviewer and checked by a second reviewer, with disagreements resolved by discussion.

Outcomes

The primary short-term treatment-success outcome was defined as a negative Dix-Hallpike test, absence of positional nystagmus, or clearly equivalent study-defined cure/resolution at Day 7 or approximately one week. A broader short-term sensitivity framework allowed the earliest objective post-treatment assessment up to one month, when no strict one-week endpoint was available. Recurrence was defined as the return of vertigo with positive Dix-Hallpike or positional nystagmus after previous successful treatment, assessed at three months or later or at the longest available follow-up. Secondary outcomes included Dizziness Handicap Inventory, Vestibular Activities and Participation Scale, vertigo intensity, vertigo frequency, treatment failure, rescue or crossover treatment, adherence, and adverse events.

Risk of Bias and Certainty Assessment

Risk of bias was assessed using the Cochrane RoB 2 framework, with the effect of interest defined as the effect of assignment to the intervention [16]. Risk-of-bias assessments were performed separately by two reviewers, with disagreements resolved by discussion and, when necessary, adjudication by a third reviewer. Judgments were made separately for short-term treatment success and recurrence across five domains: randomization process, deviations from intended interventions, missing outcome data, measurement of the outcome, and selection of the reported result. Certainty of evidence was summarized using GRADE principles for short-term treatment success, recurrence, adverse events, and continuous/functional outcomes [17].

Statistical Synthesis

For binary outcomes, risk ratios and 95% confidence intervals were calculated using arm-level event counts. For short-term treatment success, risk ratios greater than one favored Epley-type maneuvers. For recurrence, risk ratios less than one favored Epley-type maneuvers. Random-effects models were used because clinical and methodological heterogeneity was anticipated across trials. Heterogeneity was assessed using I² and τ². Binary meta-analyses were conducted using random-effects DerSimonian-Laird models, with between-study variance estimated using the DerSimonian-Laird method. Analyses were cross-checked using jamovi/MAJOR (Jonathon Love, Damian Dropmann, and Ravi Selker, Sydney, Australia) and RStudio (Posit, Boston, MA). Final forest plots were generated in RStudio using R (R Foundation for Statistical Computing, Vienna, Austria).

Sensitivity and special-handling analyses examined the influence of Zhang et al. because all study arms received balanced betahistine baseline therapy, and Hanapi et al. because its objective short-term endpoint was reported at one month [7,13]. Choi et al. was summarized separately because it enrolled patients with posterior-canal cupulolithiasis rather than typical posterior-canal canalithiasis [10]. Self-Epley and clinician-performed Epley-type maneuvers were considered narratively because the number of studies was too small for reliable subgroup analysis. Formal funnel-plot asymmetry testing and Egger’s test were not performed because fewer than 10 studies contributed to each pooled outcome, making formal assessment of small-study effects unreliable.

Results

Study Selection

**Figure 1:**
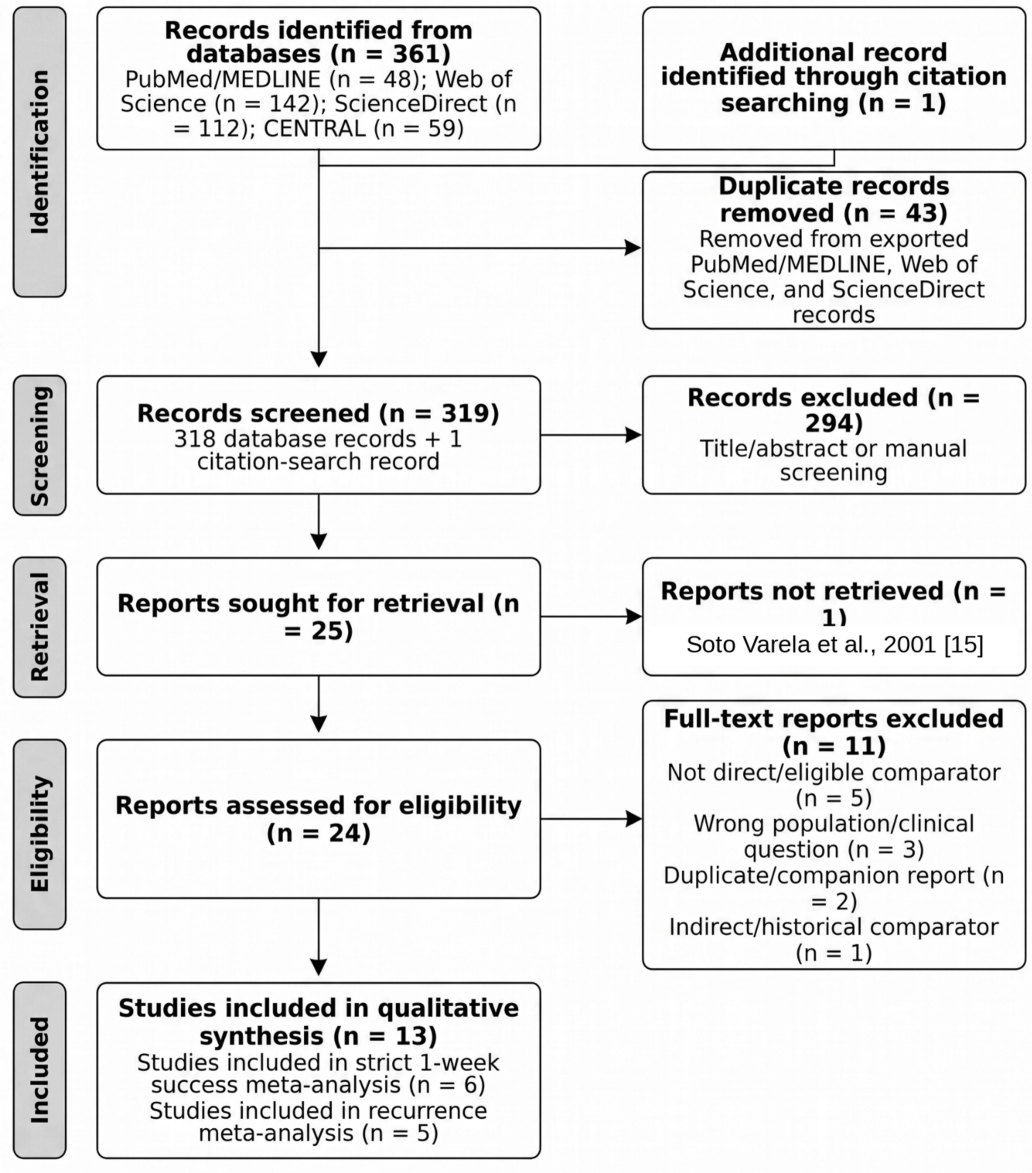
PRISMA flow diagram for the current review.

Database searches identified 361 records: PubMed/MEDLINE (n = 48), Web of Science (n = 142), ScienceDirect (n = 112), and CENTRAL (n = 59). After removal of 43 duplicates from the exported PubMed/MEDLINE, Web of Science, and ScienceDirect records, 259 exported records remained; together with the 59 manually screened CENTRAL records, 318 database records were screened. One additional record was identified through citation searching, giving 319 records screened. A total of 294 records were excluded at title/abstract or manual screening. Twenty-five reports were sought for retrieval; one report could not be retrieved, 24 full-text reports were assessed for eligibility, 11 full-text reports were excluded, and 13 studies were included in the qualitative synthesis. The study selection process is summarized in Figure 1.

Characteristics of Included Studies

Thirteen studies published between 1999 and 2026 were included, with 911 analyzed participants across all included study arms [1-13]. Studies were conducted in Germany, the United States, India, Spain, Egypt, Turkey, Mexico, South Korea, Malaysia, and China. Eligible comparisons included clinician-performed Epley/modified Epley/particle repositioning maneuvers, canalith repositioning procedures, and self-Epley or modified self-Epley maneuvers compared with Brandt-Daroff exercises. Follow-up ranged from an immediate or one-week assessment to 48 months. The characteristics of included studies are summarized in Table 1.

**Table 1:** Characteristics of included studies.

Study	Country	Design	Analyzed n/population	Eligible arms	Follow-up
Radtke et al., 1999 [1]	Germany	Prospective comparative quasi-randomized trial with partial non-random allocation	54	Modified self-Epley (MEP) n = 28; Brandt-Daroff exercise (BDE) n = 26	1 week
Cohen and Kimball, 2005 [2]	USA	Prospective randomized sham-controlled trial	124	Modified CRP n = 24; modified liberatory maneuver n = 25; Brandt-Daroff n = 25; habituation n = 25; sham n = 25	1 week, ~3 months, ~6 months; up to 210 days
Karanjai and Saha, 2010 [3]	India	Prospective randomized study	48	Semont n = 16; Epley n = 16; Brandt-Daroff n = 16	2 weeks, 1 month, 3 months
Kumar et al., 2016 [6]	India	Pre-test/post-test experimental comparative study with simple random sampling	30	Canalith repositioning procedure (CRP) n = 15; Brandt-Daroff exercises n = 15	7 days
Zhang et al., 2016 [7]	China	Randomized comparative study with control group	168	Modified Epley n = 45; modified Semont n = 43; Brandt-Daroff n = 40; control n = 40	1 week, 1 month, 6 months
Amor-Dorado et al., 2012 [4]	Spain	Randomized prospective clinical trial; masked outcome assessor	81	PRM/modified Epley n = 41; Brandt-Daroff exercise n = 40	Day 7; 1, 6, 12, 24, 36, 48 months
Abdel Kader et al., 2014 [5]	Egypt	Prospective randomized comparative study	60	Epley n = 20; rolling-over maneuver n = 20; Brandt-Daroff n = 20	Weekly after initial visit; recurrence followed for first year
Cetin et al., 2018 [8]	Turkey	Prospective randomized comparative clinical trial; successive allocation	50	Modified Epley n = 25; Brandt-Daroff n = 25	Weekly until recovery; 12-24 months recurrence follow-up (mean 18 months)
Choi et al., 2020 [10]	South Korea	Randomized clinical trial	62	Epley n = 29; Brandt-Daroff n = 33	Immediate within 1 hour; 1 week
Gupta et al., 2019 [9]	India	Randomized comparative study	90	Epley n = 30; Semont n = 30; Brandt-Daroff n = 30	Dix-Hallpike after treatment; VAP after 7 days for Epley/Semont; after 15 days for Brandt-Daroff
Celis-Aguilar et al., 2022 [12]	Mexico	Single-blind randomized controlled clinical trial	34	Brandt-Daroff n = 9; sham n = 7; Semont n = 9; Epley n = 9 after eliminations	1 week; 2-4 weeks
Hanapi et al., 2026 [13]	Malaysia	Prospective single-blind randomized controlled study	50	Self-Epley maneuver n = 25; Brandt-Daroff exercise n = 25	1 month and 6 months
Muniraju and Sangoli, 2021 [11]	India	Prospective comparative study; random assignment mentioned	60	Epley n = 30; Brandt-Daroff n = 30	1 week, 1 month, 3 months, 6 months

Risk of Bias

Risk of bias was assessed separately by outcome using the Cochrane RoB 2 framework. For the primary strict one-week treatment-success analysis, two studies were judged high risk overall, primarily because of non-random or predictable allocation, and four studies had some concerns. For the recurrence analysis, two studies were judged high risk overall because of predictable allocation or post-randomization exclusions after treatment switching; the remaining three studies had some concerns. Supportive qualitative and sensitivity studies were generally judged as having some concerns or high risk, mainly because of unclear allocation concealment, inconsistent follow-up timing, incomplete extractable outcome data, or attrition. A summary of risk-of-bias judgments is provided in Table 2, and detailed domain-level judgments are provided in the Appendices.

**Table 2:** Summary of RoB judgments. Risk of bias was assessed using the Cochrane RoB 2 framework. The effect of interest was the effect of assignment to the intervention. Detailed domain-level judgments are provided in the Appendices.

Study	Main analysis role	Overall risk of bias	Main reason for judgment
Radtke et al., 1999 [1]	Strict one-week success	High	Randomization was abandoned/partly predictable; assessor blinding was unclear.
Cohen and Kimball, 2005 [2]	Qualitative/functional outcomes	Some concerns	Randomization and blinded technicians were reported, but dropouts were replaced/excluded and clean arm-level binary data were not available for the main model.
Karanjai and Saha, 2010 [3]	Qualitative/sensitivity	Some concerns	Random assignment was reported, but randomization method, allocation concealment, blinding, and detailed outcome handling were unclear.
Amor-Dorado et al., 2012 [4]	Day 7 success and recurrence	Some concerns for short-term success; high for recurrence	Randomization and masked assessment were strong, but recurrence analysis was affected by post-randomization treatment switching/exclusions.
Abdel Kader et al., 2014 [5]	Recurrence and qualitative success	Some concerns	Randomization method, allocation concealment, and blinding were not clearly described; recurrence denominators appeared to include cured patients rather than all randomized participants.
Kumar et al., 2016 [6]	Qualitative/functional outcomes	High	Allocation and blinding were unclear; binary success counts were not cleanly extractable and outcome reporting was inconsistent.
Zhang et al., 2016 [7]	Strict one-week success and recurrence	Some concerns	Randomization was stated, but allocation concealment and assessor blinding were unclear; all groups received balanced betahistine co-intervention.
Cetin et al., 2018 [8]	One-week success and recurrence	High	Successive patient allocation made group assignment predictable, although outcome assessment used objective testing.
Gupta et al., 2019 [9]	Qualitative/functional outcomes	High	Randomization details and blinding were unclear; outcome timing differed between treatment groups.
Choi et al., 2020 [10]	Cupulolithiasis-specific qualitative/sensitivity evidence	Some concerns for immediate outcome; high for one-week outcome	Web-based randomization and blinded immediate assessment were reported, but one-week follow-up had substantial attrition and the study population was cupulolithiasis-specific.
Muniraju and Sangoli, 2021 [11]	One-week success and recurrence	Some concerns	Random assignment was mentioned, but allocation concealment, assessor blinding, and prespecified reporting were unclear.
Celis-Aguilar et al., 2022 [12]	One-week success	Some concerns	Computerized randomization and participant blinding were reported, but final groups were small and assessor blinding/selective-reporting safeguards were unclear.
Hanapi et al., 2026 [13]	Home-based short-term/long-term qualitative and sensitivity evidence	Some concerns	Block randomization, sealed envelopes, trial registration, and investigator blinding were reported; concerns related mainly to adherence and imputation.

Short-Term Treatment Success

Six studies provided extractable binary data for the primary strict one-week treatment-success meta-analysis, representing 348 participants in eligible Epley-type and Brandt-Daroff arms [1,4,7,8,11,12]. The primary short-term assessment time point was Day 7, or approximately one week. Celis-Aguilar et al. contributed their first-visit/one-week resolution data [12]. Hanapi et al. was not included in the primary strict one-week analysis because its objective Dix-Hallpike conversion endpoint was reported at one month; it was retained as a broader short-term sensitivity and qualitative study [13].

**Figure 2:**
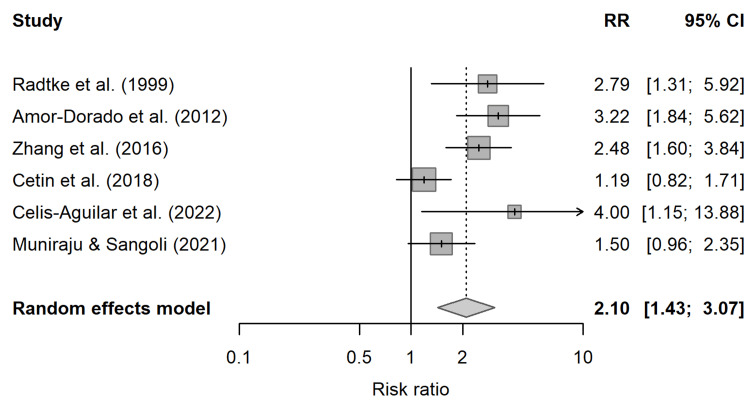
Forest plot for strict one-week success. Risk ratio >1 favors Epley-type maneuvers. Events represent treatment success at Day 7 or approximately one week. Random-effects model used. RR >1 favors Epley-type maneuvers. CI = confidence interval; RR = risk ratio

In the strict one-week analysis including six studies, Epley-type maneuvers significantly improved short-term treatment success compared with Brandt-Daroff exercises (risk ratio 2.10, 95% confidence interval 1.43-3.07; p < 0.001; I² = 65.6%; τ² = 0.1377; heterogeneity p = 0.0125; random-effects DerSimonian-Laird model). Sensitivity analyses did not materially change the direction of effect. Excluding Zhang et al., in which all arms received betahistine as balanced baseline therapy, retained a significant effect favoring Epley-type maneuvers (risk ratio 1.75, 95% confidence interval 1.19-2.58) [7]. In a broader short-term sensitivity analysis including Hanapi et al. using its one-month Dix-Hallpike conversion endpoint, the effect remained in favor of Epley-type maneuvers (risk ratio 1.86, 95% confidence interval 1.29-2.69; I² = 78.0%) [13]. The primary strict one-week treatment-success data are summarized in Table 3 and Figure 2.

**Table 3:** Short-term treatment-success meta-analysis data. BDE = Brandt-Daroff exercises; CI = confidence interval; RR = risk ratio

Study	Time point	Epley-type success	BDE success	RR (95% CI)	Notes
Radtke et al., 1999 [1]	1 week	18/28	6/26	2.79 (1.31-5.92)	High risk of bias for allocation; self-treatment subgroup.
Zhang et al., 2016 [7]	1 week	39/45	14/40	2.48 (1.60-3.84)	All arms also received betahistine; self-treatment subgroup.
Amor-Dorado et al., 2012 [4]	Day 7	33/41	10/40	3.22 (1.84-5.62)	Key randomized prospective trial; blinded follow-up assessor.
Cetin et al., 2018 [8]	1 week	19/25	16/25	1.19 (0.82-1.71)	Successive patient allocation; some concern/high risk in randomization domain.
Celis-Aguilar et al., 2022 [12]	1st visit/1 week	8/9	2/9	4.00 (1.15-13.88)	Small sample; single-blind RCT.
Muniraju and Sangoli, 2021 [11]	1 week	21/30	14/30	1.50 (0.96-2.35)	Prospective comparative study; randomization details limited.

Recurrence

Five studies contributed extractable recurrence data, representing 301 participants in the recurrence model [4,5,7,8,11]. Follow-up ranged from six months to 48 months. Recurrence definitions and denominators varied across studies; some studies reported recurrence among all randomized participants, while others reported recurrence among those with initial resolution or among those retained after treatment changes. Because recurrence denominators varied across studies, recurrence was pooled using the reported arm-level counts without additional denominator adjustment, and the finding was interpreted cautiously with supporting sensitivity analyses.

**Figure 3:**
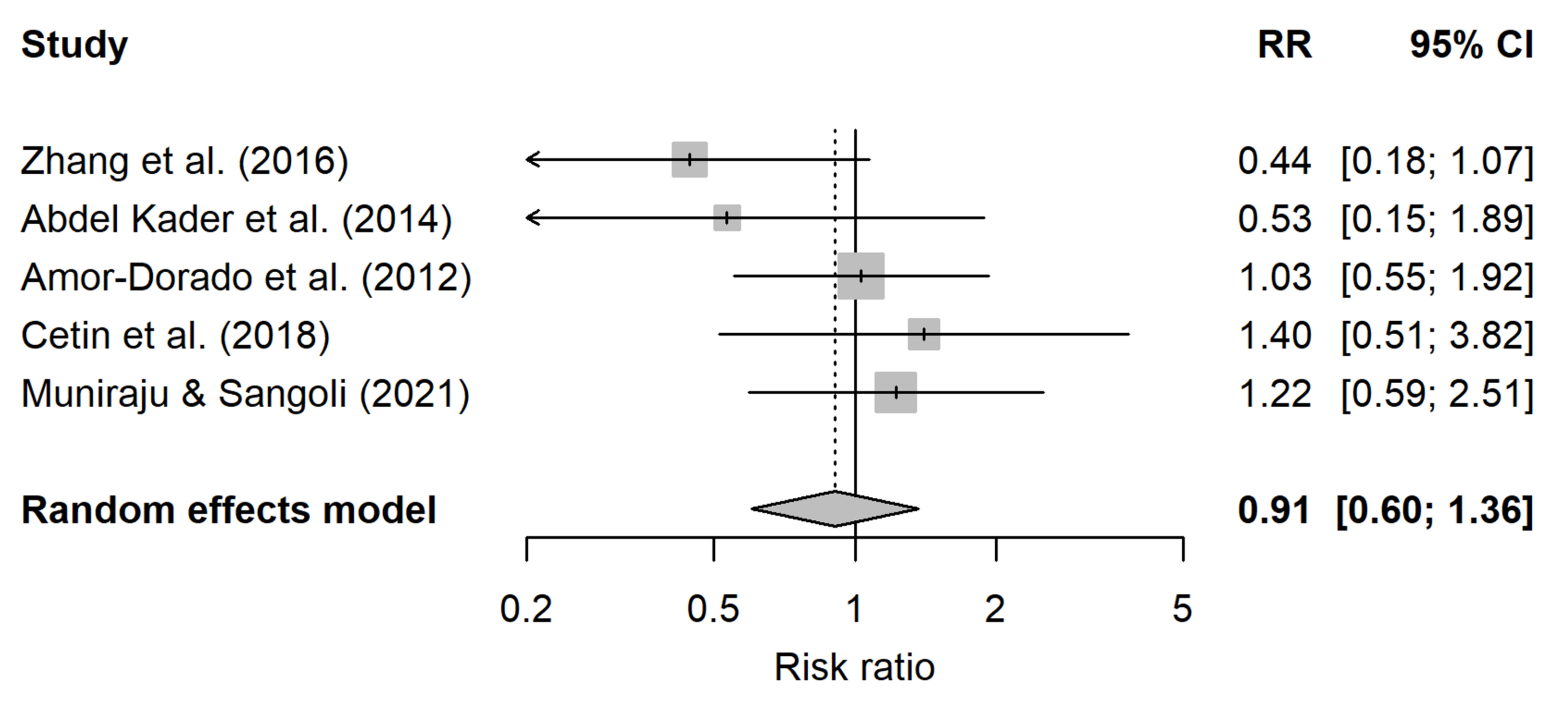
Forest plot for recurrence. Risk ratio <1 favors Epley-type maneuvers. Events represent recurrence after initial successful treatment at the longest eligible follow-up. Random-effects model used. RR <1 favors Epley-type maneuvers. CI = confidence interval; RR = risk ratio

In the recurrence analysis including five studies, Epley-type maneuvers were not associated with a statistically significant reduction in recurrence compared with Brandt-Daroff exercises (risk ratio 0.91, 95% confidence interval 0.60-1.36; p = 0.636; I² = 15.3%; τ² = 0.0336; heterogeneity p = 0.317; random-effects DerSimonian-Laird model). Long-term-only sensitivity analysis of studies with at least 12 months of follow-up was also consistent with no clear difference (risk ratio 1.00, 95% confidence interval 0.62-1.64). Excluding Zhang et al. or Muniraju and Sangoli did not change the conclusion [7,11]. The recurrence data are summarized in Table 4 and Figure 3.

**Table 4:** Recurrence meta-analysis data. BDE = Brandt-Daroff exercises; CI = confidence interval; RR = risk ratio

Study	Follow-up	Epley-type recurrence	BDE recurrence	RR (95% CI)	Notes
Zhang et al., 2016 [7]	6 months	6/45	12/40	0.44 (0.18-1.07)	Self-treatment study; all arms received betahistine; recurrence assessed by phone then positioning tests.
Abdel Kader et al., 2014 [5]	1 year	3/18	5/16	0.53 (0.15-1.89)	Recurrence denominator appears to be patients cured after initial management, not all randomized.
Amor-Dorado et al., 2012 [4]	48 months	15/41	11/31	1.03 (0.55-1.92)	BD denominator excludes 9 patients switched to PRM after month 1; recurrence after previous negative DHM.
Cetin et al., 2018 [8]	12-24 months (mean 18)	7/25	5/25	1.40 (0.51-3.82)	Recurrence during 12-24 month follow-up after weekly follow-up until recovery.
Muniraju and Sangoli, 2021 [11]	6 months	11/30	9/30	1.22 (0.59-2.51)	Recurrence reported as 36.6% vs 30%; definition stated as recurrence after previous negative DHM.

Continuous and Functional Outcomes

Continuous and functional outcomes included Dizziness Handicap Inventory, Vestibular Activities and Participation Scale, vertigo intensity, and vertigo frequency. These outcomes were not pooled because scales, assessment timing, and reporting format differed substantially. Some studies reported means and standard deviations, others reported medians or ranges, and some provided model-based estimated marginal means or incomplete variance measures. Overall, functional outcomes generally improved after treatment in both groups. Some studies suggested larger early improvements after Epley-type maneuvers, but the comparative continuous-outcome evidence remains supportive rather than definitive.

Adverse Events and Tolerability

Adverse-event reporting was inconsistent and did not support quantitative pooling. Reported events were generally minor and transient, including treatment-provoked vertigo or dizziness, nausea, vomiting, fatigue, neck pain, discomfort during maneuvers, and early discontinuation. Amor-Dorado et al. reported nausea/vomiting but no canal conversion during treatment [4]. Radtke et al. reported distressing side effects and early discontinuation in both groups [1]. Choi et al. excluded two individuals before analysis because severe vomiting prevented treatment [10]. Overall, available evidence suggests that both interventions are generally tolerated, but comparative safety is uncertain because adverse-event definitions and ascertainment were inconsistent.

Certainty of Evidence

Using GRADE, certainty was low for short-term treatment success, mainly because of the risk of bias and substantial heterogeneity. Certainty was low for recurrence because of the risk of bias and imprecision. Certainty was very low for adverse events and continuous/functional outcomes because reporting was inconsistent and data were not suitable for robust pooled analysis. The GRADE summary of findings is presented in Table 5.

**Table 5:** GRADE summary of findings.

Outcome	Effect/result	Certainty	Interpretation	Reason
Short-term treatment success (negative Dix-Hallpike/absence of positional nystagmus at Day 7 or approximately 1 week)	RR 2.10, 95% CI 1.43-3.07; I² 65.6%; τ² 0.1377	Low	Epley-type maneuvers may improve short-term treatment success compared with Brandt-Daroff, but confidence is limited by risk of bias and heterogeneity.	Started high for randomized evidence; downgraded one level for risk of bias and one level for inconsistency.
Recurrence after initial successful treatment (≥3 months / longest available follow-up)	RR 0.91 (95% CI 0.60 to 1.36); I² 15.3%; τ² 0.0336	Low	Epley-type maneuvers may not meaningfully reduce recurrence compared with Brandt-Daroff; evidence is uncertain.	Started high; downgraded one level for risk of bias and one level for imprecision.
Adverse events/tolerability	Narrative synthesis only; events were generally minor/transient (vertigo, nausea/vomiting, fatigue, neck pain, discomfort); serious adverse events rarely reported or absent.	Very low	Reported adverse events were generally minor and transient, but comparative safety remains very uncertain because adverse-event reporting was inconsistent.	Started high; downgraded for risk of bias, inconsistency in measurement/reporting, and imprecision/sparse data.
Continuous/functional outcomes (Dizziness Handicap Inventory, Vestibular Activities and Participation Scale, vertigo intensity)	Narrative synthesis only; most studies show improvement in both groups, with some early advantage for Epley-type maneuvers.	Very low	Functional outcomes improved in many studies after treatment, but comparative effect is very uncertain.	Included as supportive secondary evidence only.

Discussion

Principal Findings

This systematic review and meta-analysis found that Epley-type maneuvers were associated with higher short-term treatment success than Brandt-Daroff exercises in posterior-canal BPPV. In the primary strict one-week analysis, Epley-type maneuvers improved treatment success compared with Brandt-Daroff exercises (risk ratio 2.10, 95% confidence interval 1.43-3.07), while recurrence did not clearly differ between groups (risk ratio 0.91, 95% confidence interval 0.60-1.36). This distinction is clinically important: Epley-type maneuvers appear more effective for early resolution, but available data do not demonstrate a durable recurrence-prevention advantage.

Interpretation of Short-Term Success

The short-term result is biologically and clinically plausible. Epley-type maneuvers are designed to reposition posterior-canal otoconial debris directly, which can rapidly convert a positive Dix-Hallpike test to negative and relieve positional nystagmus. Brandt-Daroff exercises depend on repeated self-performed positional changes and habituation or gradual debris mobilization, which may produce slower improvement. Several included studies were consistent with this pattern, especially Radtke et al., Amor-Dorado et al., Zhang et al., and Celis-Aguilar et al. [1,4,7,12]. However, the magnitude of benefit varied considerably between studies, likely reflecting differences in self-treatment versus clinician-performed maneuvers, canalithiasis versus cupulolithiasis, follow-up timing, adherence, and risk of bias.

Interpretation of Recurrence Findings

The recurrence analysis showed no statistically clear difference between groups. This does not mean the treatments are identical in all long-term respects; rather, the available evidence is imprecise and heterogeneous in definitions and denominators. Recurrence may be influenced by factors beyond the initial successful maneuver, including patient-level susceptibility, vestibular comorbidity, and prior BPPV history; therefore, recurrence findings should be interpreted separately from early treatment success. Epley-type maneuvers may resolve the acute episode faster without necessarily changing the longer-term risk of recurrent symptoms.

Role of Brandt-Daroff Exercises

Brandt-Daroff exercises should not be dismissed. Although they appear less effective for rapid resolution, they remain a practical home-based option, particularly when supervised repositioning is unavailable, patients prefer self-management, or neck/back mobility limits clinician maneuvers. They may also have a role in selected patients with recurrent symptoms or as adjunctive home therapy, although adjunctive use was not the focus of the present direct-comparison review.

Continuous Outcomes and Adverse Events

Functional outcomes generally improved in both groups, but the evidence was too inconsistent for reliable pooling. Differences in Dizziness Handicap Inventory scores, Vestibular Activities and Participation scores, vertigo intensity scales, timing, and reporting format limit interpretability. Adverse events were generally minor and transient, but reporting was inconsistent. Therefore, conclusions about comparative safety should remain cautious. Future trials should use standardized adverse-event definitions and report numerator/denominator data for nausea, vomiting, canal conversion, falls, neck pain, treatment discontinuation, and exercise intolerance.

Clinical Implications

For patients with posterior-canal canalithiasis and no contraindications, Epley-type maneuvers should remain the preferred option when rapid symptom and Dix-Hallpike resolution are desired. Brandt-Daroff exercises may be reasonable when home-based treatment is needed, when recurrence self-management is being taught, or when patients cannot access or tolerate supervised maneuvers. Clinicians should counsel patients that faster early resolution does not necessarily imply lower long-term recurrence risk.

Research Implications

Future trials should use concealed randomization, blinded outcome assessment, standardized Dix-Hallpike conversion endpoints, clear recurrence definitions after documented initial resolution, and consistent follow-up intervals at one week, one month, six months, and 12 months. Trials should separate canalithiasis from cupulolithiasis and report home-exercise adherence. Reporting should include raw event counts and variance measures for continuous outcomes to support future meta-analysis.

Strengths and Limitations

Strengths of this review include a focused posterior-canal BPPV question, restriction to direct Epley-type versus Brandt-Daroff comparisons, separation of short-term success from recurrence, predefined handling of self-Epley and modified Epley maneuvers, and sensitivity analysis for studies with special design features such as balanced betahistine co-intervention or cupulolithiasis-specific enrollment.

Limitations are important. Several included studies were small and had some concerns or a high risk of bias. Randomization and allocation concealment were often incompletely described, and assessor blinding was inconsistent. Outcome definitions varied, especially for recurrence. Continuous outcomes could not be pooled robustly. Embase was not searched because institutional access was unavailable. CENTRAL was searched manually through the Cochrane Library interface, but export was not available; therefore, CENTRAL records were screened manually and not fully integrated into citation-manager deduplication. The English full-text restriction may have excluded relevant evidence.

## Conclusions

Epley-type maneuvers are associated with higher short-term treatment success than Brandt-Daroff exercises in posterior-canal BPPV. Available evidence does not show a clear reduction in recurrence with Epley-type maneuvers compared with Brandt-Daroff exercises. Reported adverse events were generally minor and transient, but comparative safety remains uncertain. The findings support Epley-type maneuvers as the preferred option for rapid resolution, while Brandt-Daroff exercises remain a reasonable home-based alternative in selected patients. Final interpretation should consider the low certainty of evidence, substantial heterogeneity in short-term success, and limitations in recurrence reporting.

## Appendices

**Table 6:** RoB 2 judgments for primary quantitative analyses. Risk of bias was assessed using the Cochrane RoB 2 framework, with the effect of interest defined as the effect of assignment to the intervention. BPPV = benign paroxysmal positional vertigo; D1 = randomization process; D2 = deviations from intended interventions; D3 = missing outcome data; D4 = measurement of the outcome; D5 = selection of the reported result; PRM = particle repositioning maneuver; VNG = videonystagmography

Study	Outcome/analysis role	RoB 2 domain judgments	Overall	Key rationale
Radtke et al., 1999 [1]	Strict 1-week success/primary	D1 high; D2 some concerns; D3 low; D4 some concerns; D5 some concerns	High	Randomization was abandoned/partly predictable and 10 participants were directly allocated to modified Epley. Outcome was objective, but assessor blinding was not clearly reported.
Zhang et al., 2016 [7]	Strict 1-week success/primary	D1 some concerns; D2 some concerns; D3 low; D4 some concerns; D5 some concerns	Some concerns	Random allocation was stated, but method, allocation concealment, and assessor blinding were unclear. All groups received balanced betahistine, so this is flagged as a co-intervention rather than a reason for exclusion.
Amor-Dorado et al., 2012 [4]	Day-7 success/primary	D1 low; D2 low; D3 low; D4 low; D5 some concerns	Some concerns	Computer-generated randomization with node assistant and masked assessor; Day-7 Dix-Hallpike/BPPV outcome was objective and complete. Protocol/analysis-plan availability was limited.
Cetin et al., 2018 [8]	1-week success/primary	D1 high; D2 some concerns; D3 low; D4 low; D5 some concerns	High	Participants were allocated by successive assignment, making allocation predictable. VNG/Dix-Hallpike outcome was objective and follow-up was complete.
Celis-Aguilar et al., 2022 [12]	1-week resolution/primary	D1 low; D2 some concerns; D3 some concerns; D4 low; D5 some concerns	Some concerns	Computerized randomization and participant blinding were reported. Four participants were lost after allocation and analyses used small final groups; assessor blinding and selective-reporting safeguards were not fully clear.
Muniraju and Sangoli, 2021 [11]	1-week success/primary	D1 some concerns; D2 some concerns; D3 low; D4 some concerns; D5 some concerns	Some concerns	Random assignment was mentioned, but allocation concealment, assessor blinding, and prespecified reporting were unclear. Outcomes were based on Dix-Hallpike testing and appeared complete.
Zhang et al., 2016 [7]	6-month recurrence/primary recurrence	D1 some concerns; D2 some concerns; D3 low; D4 some concerns; D5 some concerns	Some concerns	Randomization/concealment and blinding were unclear. Recurrence was assessed after 6 months with clinical confirmation for reported symptom recurrence, but ascertainment details were limited.
Abdel Kader et al., 2014 [5]	1-year recurrence/primary recurrence	D1 some concerns; D2 some concerns; D3 some concerns; D4 some concerns; D5 some concerns	Some concerns	Randomization method/concealment and blinding were not described. Recurrence denominators appear to be cured patients rather than all randomized participants.
Amor-Dorado et al., 2012 [4]	48-month recurrence/primary recurrence	D1 low; D2 some concerns; D3 high; D4 low; D5 some concerns	High	Initial randomization and masked assessment were strong, but 9 Brandt-Daroff patients switched to PRM after lack of improvement and were excluded from later comparative analyses.
Cetin et al., 2018 [8]	12-24 month recurrence/primary recurrence	D1 high; D2 some concerns; D3 low; D4 low; D5 some concerns	High	Successive allocation created high risk in the randomization domain. Recurrence follow-up was reported and outcome assessment used VNG/Dix-Hallpike, but allocation was predictable.
Muniraju and Sangoli, 2021 [11]	6-month recurrence/primary recurrence	D1 some concerns; D2 some concerns; D3 low; D4 some concerns; D5 some concerns	Some concerns	Random assignment was stated but concealment/blinding were unclear. Recurrence definition was described after previous negative Dix-Hallpike, but assessor masking and prespecified reporting were unclear.

**Table 7:** RoB 2 judgments for qualitative or sensitivity-only studies. Risk of bias was assessed using the Cochrane RoB 2 framework. These studies were used for qualitative synthesis, supportive interpretation, or sensitivity analyses rather than the primary pooled analysis. D1 = randomization process; D2 = deviations from intended interventions; D3 = missing outcome data; D4 = measurement of the outcome; D5 = selection of the reported result; ITT = intention-to-treat; LOCF = last observation carried forward; PI = principal investigator

Study	Outcome/analysis role	RoB 2 domain judgments	Overall	Key rationale
Cohen and Kimball, 2005 [2]	Qualitative/functional outcomes	D1 low/some concerns; D2 some concerns; D3 Some concerns; D4 low; D5 some concerns	Some concerns	Randomization and blinded technicians were reported, but 24 dropouts were replaced/excluded and no clean arm-level binary event data were available for the main RR model.
Karanjai and Saha, 2010 [3]	Composite 2-week/3-month success	D1 some concerns; D2 some concerns; D3 some concerns; D4 some concerns; D5 some concerns	Some concerns	Random assignment was reported, but randomization method/concealment, blinding, and detailed outcome handling were unclear. Endpoint combined relief at 2 weeks and absence of recurrence at 3 months.
Kumar et al., 2016 [6]	DHI/continuous outcome	D1 some concerns; D2 some concerns; D3 some concerns; D4 some concerns; D5 high	High	Simple random sampling was reported but allocation process and blinding were unclear. Binary success counts were not cleanly extractable and the Brandt-Daroff DHI table contains an apparent inconsistency.
Gupta et al., 2019 [9]	VAP and Dix-Hallpike response	D1 some concerns; D2 some concerns; D3 some concerns; D4 high; D5 some concerns	High	Random selection was stated, but concealment and blinding were unclear. Outcome timing differed between groups, increasing measurement bias for comparative effect.
Choi et al., 2020 [10]	Cupulolithiasis immediate/1-week response	Immediate: overall some concerns; 1-week: D3 high, overall high	Some concerns/high	Web-based randomization and blinded immediate assessment were reported. The 1-week outcome had 27% loss to follow-up and the population was posterior-canal cupulolithiasis, so it was not pooled with the main canalithiasis-focused analysis.
Hanapi et al., 2026 [13]	Home-based 1-month/6-month outcomes	D1 low; D2 some concerns; D3 some concerns; D4 low; D5 low	Some concerns	Block randomization, sealed envelopes, PI blinding, trial registration, and ITT with LOCF were reported. Some concerns relate to subjective adherence and LOCF imputation.
Abdel Kader et al., 2014 [5]	Earliest post-management success	D1 some concerns; D2 some concerns; D3 some concerns; D4 some concerns; D5 some concerns	Some concerns	Randomization was stated but method/concealment and blinding were unclear. Success appears to be post-management after home exercise rather than strict Day 7; canalithiasis/cupulolithiasis mix requires caution.
